# Detection of Plant Volatiles after Leaf Wounding and Darkening by Proton Transfer Reaction “Time-of-Flight” Mass Spectrometry (PTR-TOF)

**DOI:** 10.1371/journal.pone.0020419

**Published:** 2011-05-26

**Authors:** Federico Brilli, Taina M. Ruuskanen, Ralf Schnitzhofer, Markus Müller, Martin Breitenlechner, Vinzenz Bittner, Georg Wohlfahrt, Francesco Loreto, Armin Hansel

**Affiliations:** 1 Ionicon Analytik G.m.b.H., Innsbruck, Austria; 2 Institute of Ion Physics and Applied Physics, University of Innsbruck, Innsbruck, Austria; 3 Institute of Ecology, University of Innsbruck, Innsbruck, Austria; 4 Istituto per la Protezione delle Piante (IPP), Consiglio Nazionale delle Ricerche (CNR), Firenze, Italy; 5 Department of Physics, University of Helsinki, Helsinki, Finland; 6 Institut de Recherches sur la Catalyse et l'Environnement de Lyon (IRCELYON), Centre National de la Recherche Scientifique (CNRS), Villeurbanne, France; University of California Davis, United States of America

## Abstract

Proton transfer reaction-time of flight (PTR-TOF) mass spectrometry was used to improve detection of biogenic volatiles organic compounds (BVOCs) induced by leaf wounding and darkening. PTR-TOF measurements unambiguously captured the kinetic of the large emissions of green leaf volatiles (GLVs) and acetaldehyde after wounding and darkening. GLVs emission correlated with the extent of wounding, thus confirming to be an excellent indicator of mechanical damage. Transient emissions of methanol, C5 compounds and isoprene from plant species that do not emit isoprene constitutively were also detected after wounding. In the strong isoprene-emitter *Populus alba*, light-dependent isoprene emission was sustained and even enhanced for hours after photosynthesis inhibition due to leaf cutting. Thus isoprene emission can uncouple from photosynthesis and may occur even after cutting leaves or branches, e.g., by agricultural practices or because of abiotic and biotic stresses. This observation may have important implications for assessments of isoprene sources and budget in the atmosphere, and consequences for tropospheric chemistry.

## Introduction

A multitude of biogenic volatile organic compounds (BVOCs) are emitted by plants in various tissues [1]. BVOCs exert protective [2] and stress-related signaling roles [3], [4] and actively interact with large-scale atmospheric processes [5], [6]. BVOCs can be either constitutively emitted by plants [7] or induced by abiotic [8] and biotic [9], [10] stress conditions.

Mechanical damage of leaf tissues represents a critical stress to which plants are commonly exposed in nature throughout their whole life cycle. A “green leaf odor” is ubiquitously generated and promptly released by all plant species [11], [12] after membrane breakdown in response to adverse environmental conditions such as drying [13], [14], freezing [15], or herbivory outbreaks [16], [17]. Oxidation of some membrane constituents, the polyunsaturated fatty acids (linoleic acid and α-linolenic acid) initiates the biosynthesis of green leaf volatiles (GLVs) that proceeds through sequential multiple-step enzymatic reactions, catalyzed by the activity of lipoxygenases (LOX), hydroperoxide lyase (HPL), alcohol dehydrogenases (ADH) and acetyl transferases (AT), respectively leading to the production of C6-aldehydes, C6-alcohol and acetate esters [18], [19].

Several compounds constituting the GLVs blend are antimicrobial agents [20] to prevent the contamination of damaged tissue. But the emission of GLVs also plays key ecological roles in plant communities by priming inducible defense responses [21]–[23], triggering the release of other classes of volatiles [24]–[26], and coordinating the signal transduction pathway between stress perception and stress induction [27], [28].

Previous studies investigated the control of environmental factors on GLVs emission in response to stressful conditions such as elevated temperature and high light intensities [29] or after treatments with elevated ozone levels [30]. Either an increase [29] or no effect [31] on GLVs emission was reported in leaves exposed to high light intensities. In contrast, prolonged dark conditions impairing primary metabolism and the plant energetic status were shown to weaken the LOX pathway activity [32], [33]. The dependence of GLVs emission on light availability is intriguing, as a transient release of GLVs can be observed in intact leaves during transitions from light to dark [34], [35]. To date no explanation for this phenomenon has been provided.

Fast induction of dark conditions and wounding also triggers the emission of acetaldehyde [36]. Recent carbon isotope analysis indicated that wound-induced acetaldehyde burst in leaves is likely to derive from fatty acid oxidation rather than from transport from distant biosynthetic organs through the xylem flow [37]. Whether the acetaldehyde emission induced by wounding and light-dark transitions derives from fatty acid oxidation sharing the same branch reaction within the LOX pathway, or is the result of a different metabolic pathway present in leaves is still a matter of debate.

Detection of BVOCs and assessment of BVOC roles have been made possible by recent outstanding improvement of analytical techniques. Many BVOCs are detected by chemical ionization mass spectrometry virtually in real-time. Proton Transfer Reaction Mass Spectrometry (PTR-MS) has been successfully employed to monitor real-time emissions of GLVs [13], [31], [38]. Proton transfer reactions occur between H_3_O^+^ ions and all BVOCs with a proton affinity higher than water (166.5 kcal mol^−1^). However, only few masses can be selected by the quadrupole mass spectrometer in order to maintain a fast and highly sensitive response, which is a problem when suites of compounds are rapidly and transiently emitted, as in the case of GLVs.

Very recently, a novel Proton-Transfer-Reaction Time-of-Flight (PTR-TOF) instrument has been developed by combining a compact high resolution time-of-flight mass spectrometer (TOF-MS) with a PTR ion source [39]. The use of a time-of-flight detector allows complete and instantaneous detection of whole mass spectra with a time resolution of less than one second. Hence a full mass spectrum up to *m/z* = 315 is recorded by PTR-TOF, with single ions fully separated accordingly to their mass to charge (*m/z*) ratio. The time-of-flight detector also has a higher efficiency to transfer higher molecular weight ions than the quadrupole system and therefore shows strongly reduced detection limit for compounds with high mass to charge (*m*/z) ratios. PTR-TOF has a mass resolving power high resolution power (m/Δm∼4000) allowing to distinguish between isobaric ions making unambiguous identification of isobaric compounds possible by providing sum formula information.

The objective of this study was to test whether, by enhancing real-time detection through the use of PTR-TOF, the complex blend of BVOCs rapidly released upon leaf wounding or darkening could be further dissected. Specifically, we designed experiments to test the origin and time course of biosynthesis of GLVs and acetaldehyde after wounding and darkening, and to assess whether isoprene emission is sustained after mechanical stresses.

## Results

### BVOCs emission by wounding of illuminated and darkened leaves

**Figure 1:**
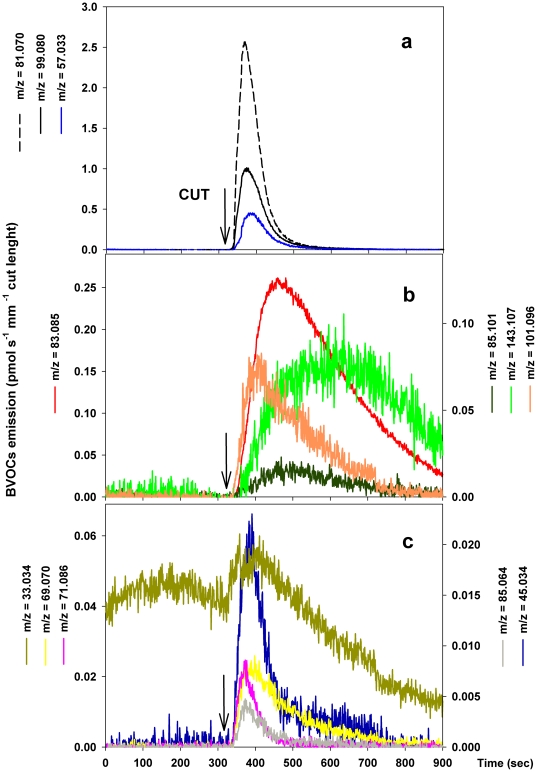
Time course of BVOCs emitted from wounded *Dactlylis glomerata* leaves PTR-TOF detected during the same measurement. Different colors and symbols indicate different ions: (a) *m/z* = 81.070+*m/z* = 99.080 [(*Z*)-3-hexenal+(*E*)-3-hexenal]; *m/z* = 57.033 (*E*)-2-hexenal; (b) *m/z* = 83.085+*m/z* = 101.096 [(*Z*)-3-hexenol+(*E*)-3-hexenol+(*E*)-2-hexenol+hexanal]; *m/z* = 85.101 [hexanol]; *m/z* = 143.107 [(*Z*)-3-hexenyl acetate+(*E*)-2-hexenyl acetate]; (c) *m/z* = 33.034 [methanol]; *m/z* = 45.034 [acetaldehyde]; *m/z* = 85.064 [pentenone]; *m/z* = 69.070 [isoprene] and *m/z* = 71.086 [pentenal fragment]. Data shown are from a single leaf but are representative of experiments replicated four times on different leaves.

Following leaf wounding a transient emission of BVOCs was induced in *Dactylis glomerata* leaves, that was thoroughly captured by PTR-TOF analysis (Fig. 1). Membrane breakdown and the consequent oxidation of unsaturated fatty acids catalyzed by the activity of LOX and HPL enzymes, result in a fast and transient evolution of C6 aldehydes. This first step of the LOX pathway was precisely detected by following the kinetic of appearance of ions with exact masses of *m/z* 81.070, 99.080, and 57.033 which represent (*Z*)- and (*E*)-3-hexenal (Fig. 1a). The initial burst of C6 aldehydes quickly decayed after peaking, since the same aldehydes are substrates for the subsequent activity of ADH. In this second step of the LOX pathway, ADH reduced hexenal and hexanal into their corresponding alcohols hexenols and hexanol, as indicated by the production of *m/z* = 83.085 and *m/z* = 85.101 in parallel with the disappearance of C6 aldehydes (Fig. 1b). The last step in the LOX pathway involves the slow conversion of C6 alcohols into hexyl- and hexenyl- acetates as a result of a further reaction catalyzed by the AT enzymes, Consistently, the appearance of *m/z* 143.107 (hexenyl acetates) was observed in the PTR-TOF spectrum (Fig. 1b).

The fast release of other volatiles, namely pentanol (as fragment C_5_H_11_^+^ at *m/z* = 71.086) and pentenone (*m/z* = 85.064), acetaldehyde (*m/z* = 45.034), and a burst of methanol (*m/z* = 33.034) exceeding the detected constitutive emission, were also detected after wounding (Fig. 1c). Finally, a transient emission of a protonated C_5_H_8_ compound with *m/z* = 69.070 that could be assigned to isoprene was observed after wounding.

**Figure 2:**
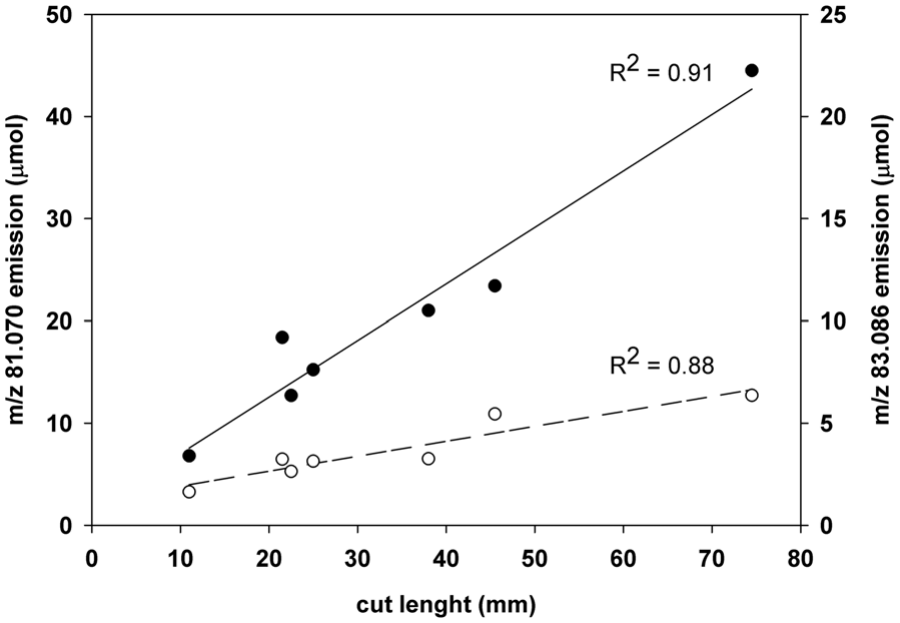
Total amount (mass) of ions *m/z* = 81.070 [(*Z*)-3-hexenal+(*E*)-3-hexenal] (black circles) and *m/z* = 83.085 [(*Z*)-3-hexenol+(*E*)-3-hexenol+(*E*)-2-hexenol+hexanal] (open circles) as a function of cut lengths [mm] produced when wounding *Dactlylis glomerata* leaves (R^2^ = 0.91 solid line; R^2^ = 0.88 dotted line). Circles represent results from 7 experiments carried out on single leaves.

The standardized conditions of our experiments allowed investigating whether the emission of wound-induced BVOC is proportional to the extent of the foliar injury. Indeed, a linear correlation between the cut length of wounding and the emission of the most representative GLVs, at *m/z* 81.070 (hexenals) and *m/z* 83.086 (hexenols and hexanal) was found (Fig. 2). Thus, GLVs represent an accurate proxy of the mechanical injury and a realistic indication of membrane deterioration [14], [30], [40].

**Figure 3:**
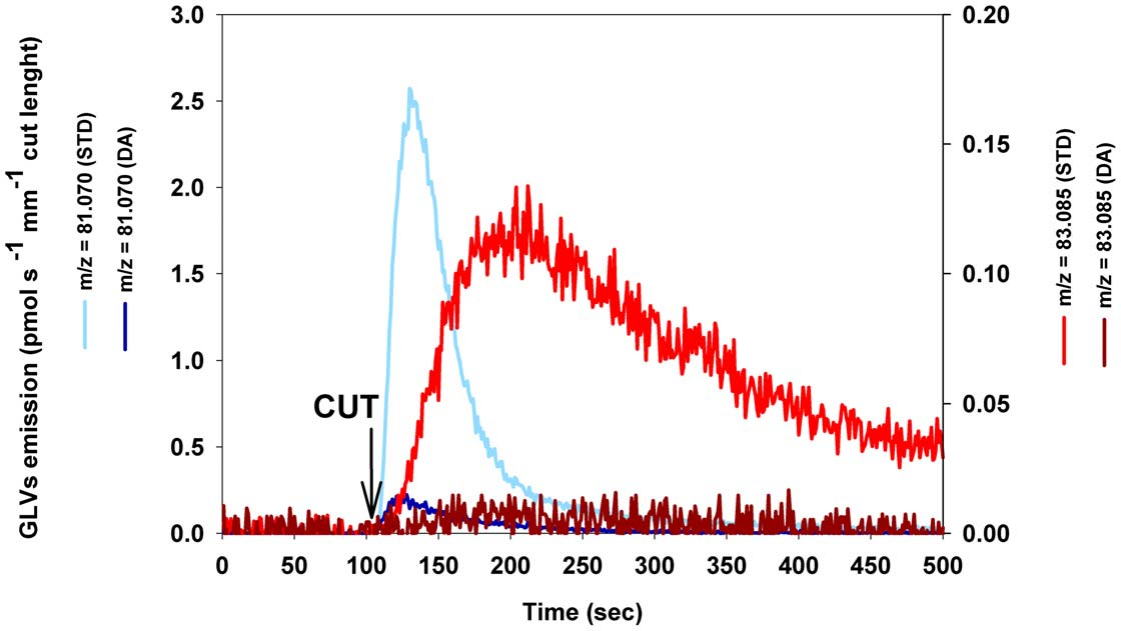
Release of GLVs after wounding *Dactlylis glomerata* plants kept in dark conditions (24 hours/day) for 7 days long (DA) compared to *Dactlylis glomerata* plants kept under standard circadian rhythm (12 hours light+12 hours dark) (STD). Different colors indicate different ions: *m/z* = 81.070 [(*Z*)-3-hexenal+(*E*)-3-hexenal]; *m/z* = 83.085 [(*Z*)-3-hexenol+(*E*)-3-hexenol+(*E*)-2-hexenol+hexanal]. Data shown are from a single leaf but are representative of experiments replicated four times on different leaves.

A few minutes of dark adaptation, as well as exposure to different light intensities before wounding did not affect either the amount or the relative abundance of the GLVs emitted (data not shown). This observation indicates that the GLVs emission was not limited by photochemical reactions, since the reducing power production stops very rapidly after the induction of dark conditions. A significant reduction of the emission of GLVs was detected only when the leaves were exposed to prolonged and continuous dark conditions (Fig. 3). PTR-TOF analysis allowed to identify different blends of GLVs released also from *Dactlylis glomerata* and *Populus alba* wounded leaves (Table 1). In both species, GLV emission was quenched when wounding leaves that were previously darkened. Interestingly, the GLV blend released by *P. alba* wounded leaves showed no substantial qualitative variations even when leaves were exposed for several days to continuous dark conditions (Table 1). Whereas, in *D. glomerata* leaves the fraction of hexyl-acetate (m/z 43.018) was larger and hexenal (m/z 81.070+83.086) was lower when wounding occurred after continuous darkening. Our results therefore indicate a species-specific sensitivity of GLVs emission to long term variation of light conditions.

**Table 1:** Total amount of green leaves volatiles (GLVs) (µmol mm^−1^) and percentage (%) of the major ions representing the GLVs blend emitted after wounding *Dactlylis glomerata* and *Populus alba* leaves grown in normal circadian light cycle compared to those exposed to continuous dark conditions for 7 days.

m/z (%) --------- plant species	43.018 (hexyl acetate)	43.054 (hexanol+hexyl acetate)	57.033 (hexanals)	61.028 (hexyl acetate)	81.070 (hexenal)	83.086 (hexenols+hexanal)	85.064 (pentenone)	85.101 (hexanol)	143.107 (hexenyl acetates)	Total amount of GLVs (µmol mm^−1^)
*Dactlylis glomerata*	4.8^b^ (±1.1)	2.6^ab^ (±1.5)	3.1^a^ (±1.8)	1.3^b^ (±0.3)	57.8^b^ (±5.2)	18.0^a^ (±1.7)	0.3^a^ (±0.05)	0.1^a^ (±0.01)	11.6^a^ (±2.0)	1.370^cd^ (±0.281)
*Dactlylis glomerata* (dark adapted)	32.4^a^ (±7.6)	5.7^a^ (±1.2)	0.5^a^ (±0.2)	11.4^a^ (±1.6)	38.0^c^ (±5.1)	3.5^c^ (±0.6)	0.4^a^ (±0.2)	0.4^a^ (±0.2)	7.9^ab^ (±2.3)	0.247^c^ (±0.047)
*Populus alba*	2.1^b^ (±0.3)	0.4^bc^ (±0.02)	2.3^a^ (±0.8)	0.07^b^ (±0.04)	78.0^a^ (±3.3)	12.4^b^ (±1.1)	0.2^a^ (±0.03)	0.04^a^ (±0.01)	4.5^ab^ (±2.0)	11.107^a^ (±1.102)
*Populus alba* (dark adapted)	0.8^b^ (±0.4)	0.3^bc^ (±0.2)	3.0^a^ (±0.6)	1.2^b^ (±0.5)	84.3^a^ (±0.6)	8.8^b^ (±0.9)	0.3^a^ (±0.1)	0.1^a^ (±0.005)	1.2^b^ (±0.5)	4.025^bd^ (±0.873)

Means ± SE are shown (*n* = 4). Differences between means within the same column were statistically assessed with a Tukey's post hoc test (*P*<0.05).

In another experiment we shaded only a part of the plant while illuminating the rest of it and confirmed that the lower emission of wound-induced GLVs was localized only in the shaded leaves, thus excluding the implication of any internal plant signaling system (data not shown).

### BVOCs emission during light-dark transitions

A rapid release of GLVs, together with a burst of acetaldehyde, were observed during previous experiments using the PTR-MS technology when exposing leaves to rapid light to dark fluctuations [35], [36]. We used the fast and highly sensitive PTR-TOF to further characterize this response in plant species having different constitutive emission.

**Figure 4:**
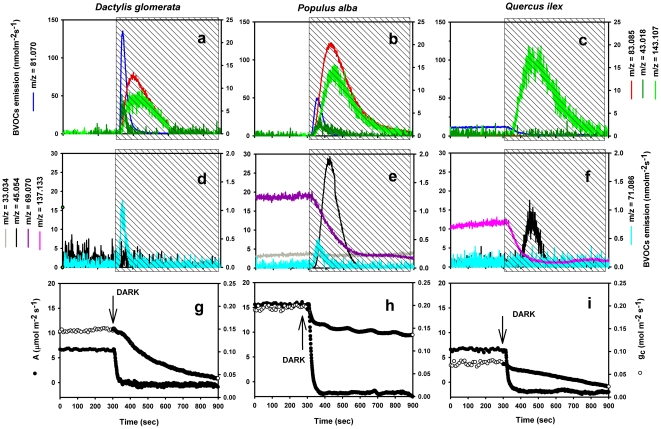
Time course of BVOC emission and gas exchange of intact *Dactlylis glomerata* (a, d, g), *Populus alba* (b, e, h) and *Quercus ilex* (c, f, i) leaves following rapid light-dark transitions. The light was switched off at the time indicated by the arrows. Different colors indicate different ions: (a, b, c) *m/z* = 81.070 [(*Z*)-3-hexenal+(*E*)-3-hexenal]; *m/z* = 83.085 [(*Z*)-3-hexenol+(*E*)-3-hexenol+(*E*)-2-hexenol+hexanal]; *m/z* = 43.018 [hexyl acetates]; m/z = 143.107 [(*Z*)-3-hexenyl acetate+(*E*)-2-hexenyl acetate]. (d, e, f) *m/z* = 33.034 [methanol]; *m/z* = 45.054 [acetaldehyde]; *m/z* = 69.070 [isoprene] (only in *P. alba -* panel e); *m/z* = 71.086 [pentenal fragment]; *m/z* = 137.133 [monoterpenes] (only in *Q. ilex -* panel f). (g, h, i) Photosynthetic carbon assimilation (black circles) and stomatal conductance (open circles). One typical sequence out of four independent experiments is shown.

**Figure 5:**
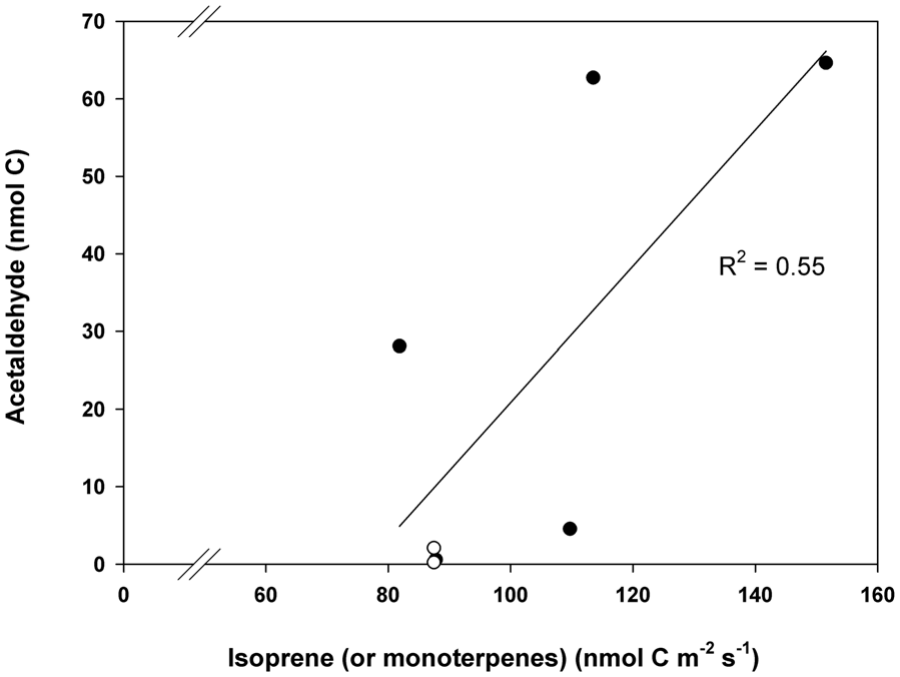
Relationship between constitutive carbon emitted as isoprenoids (isoprene in *P. alba*; monoterpenes in *Q. ilex*) and the total amount of carbon emitted as acetaldehyde after fast transition from light to dark conditions (linear regression R^2^ = 0.55). The emission measured in 3 different leaves of *P. alba* (black circles) and 4 different leaves of *Q. ilex* (white circles) are shown. Each leaf was selected from different individuals in both plant species.

In *D. glomerata* only a burst of GLVs (Fig. 4a) but no acetaldehyde emission (Fig. 4d) was measured after light-dark transitions. The transient emission of GLVs was characterized first by a strong and sharp peak of hexenals (ion *m/z* = 81.070), with smaller peaks attributable to fragments of hexyl acetate (*m/z* = 43.018) and pentanol (*m/z* = 71.086); sequentially the emissions of hexenols and hexanal (ion *m/z* = 83.085) and hexenyl acetate (ion *m/z* = 143.107) were observed (Fig. 4a). In *P. alba*, light-dark transitions triggered the burst of GLVs (Fig. 4b), but the emission was associated with a strong release of acetaldehyde (*m/z* = 45.054) and a drop of isoprene emission (*m/z* = 69.070) (Fig. 4e) as shown by Graus *et al*. (2004) [35]. *Q. ilex* showed a constitutive monoterpene emission in illuminated leaves, as measured by both the intensities of the fragment *m/z* = 81.070 (Fig. 4c) and of the protonated molecular ion *m/z* = 137.133 (Fig. 4h). Monoterpene emission declined quickly after the light was switched off, whereas, similarly to *P. alba*, a burst of GLVs (but only hexenyl acetate, Fig. 4g) and acetaldehyde (Fig. 4f) was also observed in oak leaves upon darkening. The complete drop of photosynthesis and the reduction of stomatal conductance induced by darkening are also presented for the single species (see Fig. 4, g, h, i). Finally, light-dark transition did not stimulate any BVOCs emission in the leaves of the monoterpene-storing species *C. limon* (data not shown). The constitutive level of emitted isoprenoids (isoprene by poplar or monoterpenes by oak) was directly associated to the maximum value of acetaldehyde detected during the burst (Fig. 5). When comparing the total BVOC emitted by the three species, the emissions of the three species were significantly different. The highest emission was observed in *P. alba* and the lowest in *Q. ilex* (Table 2). In any case, the emission of GLV after the light-dark transition was 3 to 4 order of magnitude lower than that of BVOCs measured after wounding (compare Tables 1 and 2). When considering the blend characteristics, the emission of *P. alba* leaves after darkening was characterized by a high percentage of hexenols + hexenal (*m/z* = 83.085). On the other hand, half of the low GLV emission of *Q. ilex* was made of hexenyl acetates (*m/z* = 143.107).

**Table 2:** Total amount of BVOCs emitted (µmol m^2^) and relative percentage (%) of the major ions representing the BVOCs blend emitted after transition from light to dark conditions in *Dactlylis glomerata*, *Quercus* ilex, *Populus alba* intact leaves and in *Populus alba* leaves after the petiole has been excised.

m/z (%) --------- plant species	43.018 (hexyl acetate)	43.054 (hexanol+hexyl acetate)	45.034 (acet aldehyde)	57.033 (hexanals)	61.028 (hexyl acetate)	81.070 (hexenal)	83.086 (hexenols+hexanal)	85.064 (pentenone)	85.101 (hexanol)	143.107 (hexenyl acetates)	Total amount of BVOCs (µmol m^−2^)
*Dactlylis glomerata*	10.3^a^ (±5.6)	0.4^b^ (±0.1)	0.6^b^ (±0.04)	1.9^b^ (±0.4)	2.8^b^ (±1.0)	35.1^a^ (±1.0)	24.0^c^ (±1.6)	1.1^a^ (±0.05)	0.6^b^ (±0.05)	23.4^b^ (±4.5)	0.0443^b^ (±0.0054)
*Populus alba*	0.9^b^ (±0.5)	0.5^b^ (±0.2)	17.8^a^ (±3.9)	2.1^b^ (±0.2)	0.5^b^ (±0.4)	17.0^b^ (±0.7)	32.7^b^ (±3.3)	0.5^b^ (±0.2)	0.6^b^ (±0.1)	33.0^b^ (±3.6)	0.0856^a^ (±0.0152)
*Populus alba* (petiole excised)	7.0^a^ (±0.3)	5.8^a^ (±1.9)	3.3^b^ (±0.5)	2.4^b^ (±0.3)	15.9^a^ (±1.7)	11.0^b^ (±2.3)	44.3^a^ (±0.6)	1.1^a^ (±0.05)	0.6^b^ (±0.2)	8.8^c^ (±0.4)	0.0351^b^ (±0.0029)
*Quercus ilex*	6.2^a^ (±1.1)	3.7^a^ (±1.1)	6.2^b^ (±2.7)	11.4^a^ (±4.1)	4.5^b^ (±4.1)	12.8^b^ (±2.2)	1.1^d^ (±0.4)	1.6^a^ (±0.3)	1.9^a^ (±0.2)	50.7^a^ (±3.9)	0.0132^b^ (±0.0016)

Means ± SE are shown (*n* = 4). Differences between means within the same column were statistically assessed with a Tukey's post hoc test (*P*<0.05).

**Figure 6:**
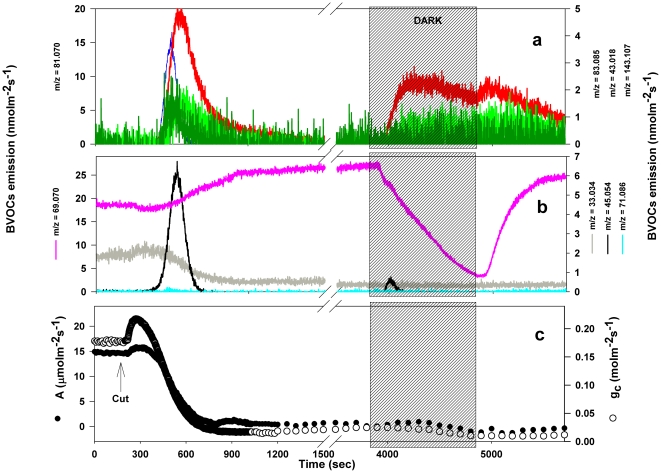
Effect of cutting on BVOCs emission (a), (b) and gas exchange (c) of a *Populus alba* leaf. At the time indicated by the arrow, the leaf petiole was excised and the light was switched off during the time indicated by the striped background. Different colors indicate different ions: (a) *m/z* = 81.070 [(*Z*)-3-hexenal+(*E*)-3-hexenal]; *m/z* = 83.085 [(*Z*)-3-hexenol+(*E*)-3-hexenol+(*E*)-2-hexenol+hexanal]; *m/z* = 43.018 [hexyl acetates]; *m/z* = 143.107 [hexenyl acetates]. (b) *m/z* = 33.034 [methanol]; *m/z* = 45.054 [acetaldehyde]; *m/z* = 71.086 [pentenal fragment]; *m/z* = 69.069 [isoprene]. (c) Photosynthetic carbon assimilation (black circles) and stomatal conductance (open circles). One typical sequence out of four independent experiments is shown.

In a separate experiment, the effect of a light-dark transition on BVOC emission was further investigated in *P. alba* leaves one hour after the leaf petiole had been cut and therefore no emission from photosynthesis intermediates was possible. The simple petiole excision triggered a transient emission of GLVs (Fig. 6a) and acetaldehyde (Fig. 6b), confirming previous observations [29], [35]. Qualitatively, *P. alba* emission was again principally made by hexenols + hexenal (*m/z* = 83.085), but the fraction of these compounds was even higher than when the transition occurred in intact leaves (Table 2). The time course recorded by PTR-TOF temporally resolved the GLV emissions and again showed a sequential increase of the *m/z = *81.070, 83.085, 43.018 and 143.107 indicating the activation of the whole lipoxygenases pathway (Fig. 6a). A rapid increase in stomatal opening after cutting, known as Ivanov effect [41], was accompanied by a transient enhancement of photosynthesis which slightly anticipated the emission of GLVs and acetaldehyde (Fig. 6c). Isoprene emission was also stimulated after leaf cutting (Fig. 6b). Interestingly, isoprene emission uncoupled from photosynthetic carbon assimilation. Isoprene emission started to increase when the constitutive emission of methanol started to decrease (Fig. 6b) and the stomatal conductance reached half of the rate of uncut leaves (Fig. 6c).

When no photosynthetic carbon was assimilated (i.e. one hour after the petiole was excised), a light-dark transition caused only a small peak of acetaldehyde and a quantitatively reduced, yet long-lasting release of GLVs, represented mainly by *m/z* 83.085 (hexenols and hexanal). Isoprene dropped upon darkening of cut *P. alba* leaves (Fig. 6b), but the rate at which isoprene was reduced was ten times slower than observed when darkening attached leaves. When the cut leaves where again illuminated, isoprene emission increased again reaching a rate similar to that observed before darkening.

### Analysis of GLVs kinetics after wounding and during light-dark transitions

**Figure 7:**
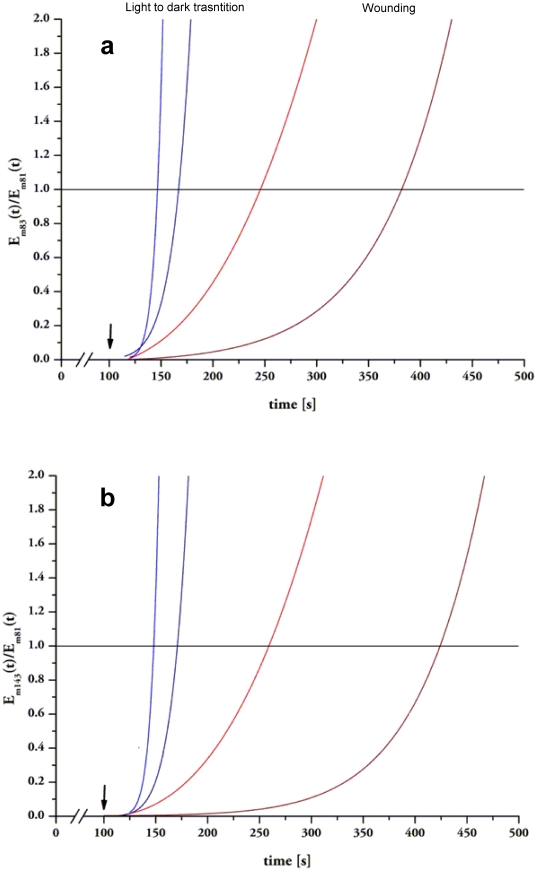
Ratios between the normalized emission rates E_k_ (t) varying over the time. Different panels indicate ratios of *m/z* = 83.085/*m/z* = 81.070 (a) and *m/z* 143.107/*m/z* 81.070 (b) emitted after cutting leaves of *Dactlylis glomerata* (red lines) and of *Populus alba* (dark red lines) or after exposing to light to dark transition leaves of *Dactlylis glomerata* (blue lines) and of *Populus alba* (dark blue lines).

*In vivo* information on the enzymatic activity leading to GLVs production cannot be provided according to Michealis-Menten model due to difficult estimation of substrate availability. This is the consequence of the continuous conversion of enzymatic products into further substrates which occurs once the cascade of multi-step enzymatic reaction (catalyzed by LOX, HPL, ADH and AD) has been initiated. However, by exploiting PTR-TOF highly resolved GLVs analysis, it may be possible to indirectly analyze differences in the activation of the lipoxygenase pathway by following the rate of conversion over the time between ratios of protonated ions. In particular the time course of the ratios between *m/z* = 83.085/81.070 (hexenols + hexanal/hexenals) and between *m/z* = 143.107/81.070 (hexenyl acetates/hexenals) may provide information about the speed of conversion of the classes of GLVs. We performed this analysis on GLVs emitted after cutting and after transition from light to dark transition by *D. glomerata* and *P. alba* leaves (Fig. 7 a,b). Our results show that wounding induces a differential production of the main GLVs over the time with respect to light-dark transition in both plant species. The instantaneous oxidation of linoleic and α-linolenic acids occurring when wounded cellular membranes are exposed to the air contact efficiently catalyzed the first step of the lipoxygenase reactions leading to the production of *m/z* = 81.070 (hexanal/hexenals) that is slowly converted to *m/z* = 83.085 (hexenols) and then to *m/z* = 143.107 (hexenyl acetates); differently the condition created by sudden darkening induces a production of both *m/z* = 81.070 than *m/z* = 83.085 (or *m/z* = 143.107) with similar time-courses.

Indeed, after darkening, *m/z* = 81.070 is released at the same rate than *m/z* = 83.085 (Fig. 7a) and *m/z* 143.107 (Fig. 7b) in *D. glomerata* and in *P. alba* leaves indicating that the enzymes implied in different steps of the lipoxygenase pathway are activated at same pace. Differently, wounding triggers a conversion of *m/z* = 81.070 into *m/z* = 83.085 (Fig. 7a) followed by *m/z* 143.107 (Fig. 7b) which was slower in *P. alba* than in *D. glomerata*, thus indicating a diverse lag time for the activation of the enzymes characterizing the second step of the lipoxygenase pathway in the two plant species.

## Discussion

Wounding and darkening have been shown to induce the emission of a suite of volatile organic compounds whose biosynthesis and ecological significance is largely unknown. The PTR-TOF allowed us to follow rapid transients of multiple compounds, and therefore demonstrated to be a promising technique to further dissect the origin of induced BVOC emissions.

**Wounding** induced a blend of GLVs, as previously reported by using different techniques [29], [32]. However, PTR-TOF also revealed:

a. *the emission of C5 compounds, such as pentenone and pentanol*. These compounds may be produced by LOX enzymes branch reactions [42] as suggested by their fast appearance after wounding, matching the kinetic of emission of aldehydes. PTR-TOF clearly resolved the detected compound (C_5_H_11_^+^) at *m/z* = 71.086 corresponding to pentanol fragment and clearly resolved it from methyl vinyl ketone (MVK) and methacrolein (MAC) having the same protonated nominal mass weight but a different protonated exact mass weight of *m/z* = 71.049 (C_4_H_7_O^+^).

b. *A burst of methanol exceeding the detected constitutive emission*. This burst may be due to the evaporation of an internal aqueous pool released by membrane breakdown. The existence of an internal methanol reservoir in leaves is consistent with the physico-chemical properties of this compound, since its low partition coefficient between gaseous and liquid phase (0.46 Pa m^3^ mol^−1^) enables it to build up an aqueous pool in the leaf [43].

c. *A small emission of isoprene in a grass that does not emit isoprene constitutively*. We speculate that such a small isoprene emission in *D. glomerata* leaves could originate from a non-enzymatic reaction due to acid hydrolysis of the DMAPP (dimethylallyl pyrophosphate) precursor after wounding, as already shown *in vitro* [44], [45]. In the case of wounding the chloroplastic DMADP may come into contact with the more acid cytosolic pH therefore initiating a limited non-enzymatic production of isoprene.

d. *A sustained and enhanced emission of isoprene in strong isoprene-emitters after the leaf was totally severed from the plant*. To our knowledge the sustained emission of isoprene after leaf cutting was never recorded before. A burst of isoprene emission was reported in wounded *Phragmites australis* leaves [29] but the emission dropped back to very low value (about one tenth of the emission reported here) within less than 10 min. Loreto and Sharkey (1993) [46] showed a sustained emission of isoprene for at least 30 min in the terminal leaflet of a trifoliated bean leaf in which a lateral leaflet was severed, but both isoprene emission and photosynthesis dropped about 10% in that case. In our experiments isoprene emission was enhanced for about 1 h after completely severing the leaf from the plant and in absence of photosynthesis. Isoprene emission can be restored even after a long darkening of the cut leaf. Enhanced isoprene emission could be sustained by a higher isoprene synthase activity, in turn stimulated by a rising internal leaf temperature consequent to the progressive stomata closure. Certainly carbon sources alternative to photosynthesis are used to sustain isoprene emission in this case. The activation of alternative carbon sources in response to severe environmental stresses has been observed [8], [47] but not in the case of mechanical stresses. No ecological reason is known so far that can explain why isoprene emission remains high after leaf cutting.

e. *A different GLVs blend in the four plant species used in our experiment*, that can be attributed either to a different composition of cellular membranes fatty acids [18], or to a differential activation of the lipoxygenase pathway in herbaceous and in woody species. The different profile of GLVs may have relevant consequences in plant-herbivore communication since herbivores perceive variations of volatile blends when discriminating between host and non-host plants [48], [49]. Host-plant recognition by herbivores can even rely on changes in GLVs isomers ratios [23].

Our experiments also show that the blend of emitted compounds is different in response to different elicitors (wounding or light-dark transitions), and may change in plants subjected to a combination of these events. It is therefore suggested that the GLV-based capacity of plants to interact with other organisms may be strongly modulated by environmental conditions.

f. *A dependence of GLVs emission from the extent of the damaged leaf blade*, confirming experiments by Fall *et al*. (1999) [31] and unambiguously inferring that GLVs are excellent indicators of mechanical damage to cellular membranes [8], [29].

g. *A general impairment of the overall lipoxygenase pathway producing GLVs after prolonged dark adaptation*. A light sensitivity of LOX and ADH enzymes activity was reported by Hatanaka (1993) [32] in cultured alfalfa green cells adapted for some days to dark conditions. We reported the same effect *in vivo* and at whole plant level in *D. glomerata* leaves maintained in dark conditions for 7 days. Despite the lower emission of hexenals (*m/z* = 81.070) and hexenols and hexanal (*m/z* = 83.085) in darkened leaves as compared to control leaves grown under a standard circadian light regime, the ratio between the two ions remained unchanged, indicating a coordinated down-regulation of LOX and AHD enzymes triggered by α-linolenic and linoleic acids substrate limitation. These substrates that can be readily catabolized to support the leaf metabolism when photosynthetic carbon is no longer assimilated [50], [51].

**Darkening** is also able to induce a temporary burst of GLVs and acetaldehyde [29], [35]. Sudden transitions from light to dark conditions trigger changes in extracellular and/or intracellular leaf pH accompanied by changes in the membranes stability [52]. As a consequence, the transient release of GLVs could represent a sensitive response of cellular membranes to arising stress conditions caused by rapid pH variation occurring during strong light fluctuations [53]. Analysis of the high time resolved GLVs kinetic, as recorded by PTR-TOF, indicated that wounding and darkening catalyze the activation of the same enzymes implied in different steps of the lipoxygenase pathway, but also suggested that the activation occurred faster in response to light-dark transition, probably because of the lower pool of GLVs that was formed.

As for acetaldehyde, this volatile may transiently increase in the leaves as a product of the pyruvate overflow pathway, when dark conditions are suddenly imposed [36]. Our experiments showed that acetaldehyde is mainly emitted after darkening isoprenoid-emitting leaves, and that the constitutive level of emitted isoprenoids (isoprene by poplar or monoterpenes by oak) may be associated to both the maximum value than to the total amount of acetaldehyde detected during the burst. Acetaldehyde emission is partially labeled by ^13^CO_2_, inferring that both a chloroplastic and a cytosolic source of carbon contribute to its formation [36]. We surmise that acetaldehyde released after darkening is formed by recycling part of carbon that generates isoprene, probably mediated by pyruvate shuttle [46]. This hypothesis, involving a previously unknown cross-talk between BVOCs, should be thoroughly tested with biochemical and molecular tools. The emission of acetaldehyde was also observed when the light-dark transition was imposed to cut leaves. However, in this case acetaldehyde emission was tiny and the release of GLVs was also considerably lower. Clearly, there have been changes imposed by leaf cutting that prevented the acetaldehyde pool from being constituted. Possibly this has to do with the slower inhibition of isoprene emission that was also observed when darkening cut leaves (Fig. 6). If isoprene emission remains sustained in darkened leaves then less carbon is available for recycling through acetaldehyde. The low emission of acetaldehyde could be also attributed to stomatal closure. Emission of volatiles with low gas-liquid phase partition coefficients is controlled by stomatal aperture [43] and therefore a tight stomatal closure, such as the one occurring in cut leaves, might uncouple synthesis and emission of acetaldehyde and GLV. Finally, changes in foliar pH may also have curbed the emission of these compounds. The emission of acetaldehyde and GLV is regulated by fast and large pH changes [53]. Perhaps foliar pH already changed in cut leaves because of photosynthesis inhibition during drying. The alkalinization of sap and apoplastic pH in drought-stressed leaves has been reported [54]. The susbsequent darkening could have caused a pH variation too small to trigger acetaldehyde and GLV production in a more alkaline apoplast.

In conclusion PTR-TOF analysis has allowed to improve measurements of complex mixtures of volatiles. Specifically, when examining BVOC emission induced by wounding, a new class of C5 compounds has been discovered, and a long-lasting enhancement of isoprene emission in cut leaves has been reported. When examining BVOC emission following a light-dark transition, a relationship between isoprene and acetaldehyde has been found, indicating the possible use of common precursors by different volatiles. Ultrafast and complete time-of-flight detection also unambiguously assigned the GLVs mixture induced by wounding or darkening, and further work may allow to use the time-course of GLVs induction as an in-vivo marker of enzyme activation and substrate availability which may be species-specific and controlled by environmental factors.

## Materials and Methods

### Plant material

Potted plants of *Populus alba* L. (a perennial isoprene emitting species), *Quercus ilex* L. (a perennial monoterpene-emitting species), and *Citrus x limon* (L.) Burm. (a perennial monoterpene-storing species) and *Dactlylis glomerata* L. (a herbaceous non-emitting isoprenoid species) were grown in the Botanical Garden greenhouse facilities of the University of Innsbruck (Innsbruck, Austria).

### Gas exchange measurements

Leaves were carefully clamped in a 200 ml gas exchange cuvette designed for conifer needles measurements (6400-05 Conifer Chamber; Li-Cor, Lincoln, NE, USA) and flushed with an air flow previously deprived from any VOC contaminants by passing through a catalytic converter kept at a constant temperature of 350°C. The air flow entering the cuvette was maintained to 2 L/min by using a mass flow controller (Bronkhorst, High-Tech B.V., Ruurlo, Netherlands) in order to have a fast and complete cuvette washout of only 6 seconds simulating the turbulent conditions occurring in the real environment.

The cuvette was connected to a portable gas exchange system (LI-6400; Li-Cor, Lincoln, NE, USA). Temperature was continuously monitored during all the measurements and ranged between 26–30°C.

For wounding experiments, the set up was modified by inserting a blade inside the cuvette connected by a small metal nut to a Teflon stripe protruding out of the cuvette. Once the leaves were properly enclosed, wounds were quickly and sharply produced by simply pulling out the Teflon stripe without opening the cuvette and therefore avoiding any contamination by ambient VOCs.

Photosynthetic carbon assimilation rate (A) and stomatal conductance (g_c_) were measured by the LI-6400 system, and BVOCs were analyzed by diverting part of the outflow air exiting the cuvette to a PTR-TOF system.

### PTR-TOF-based BVOC measurements

The proton transfer reaction-time of flight mass spectrometer (PTR-TOF) developed by the University of Innsbruck [39] and commercially available by Ionicon Analytik GmbH (Innsbruck, Austria) was used. The basic instrumental set up resembles the PTR-MS one [38], [55], but PTR front part consisting of a hollow cathode ion source and the drift tube has been interfaced with a high mass resolution, orthogonal acceleration, reflectron time-of-flight mass spectrometer TOF-MS (Tofwerk AG, Switzerland). Similarly to PTR-MS, a high concentration of reagent ions H_3_O^+^ is produced in the hollow cathode by extracting the headspace of a water reservoir. After being produced, the reagent ions H_3_O^+^ flow through the drift tube section where the gas sample is continuously injected via a precise inlet system. Proton transfer reactions occur between H_3_O^+^ ions and all the biogenic or anthropogenic VOCs having a proton affinity higher than that of water (165.2 Kcal mol^−1^) and are performed in a reaction chamber (drift tube) under controlled conditions of pressure (2.2–2.4 mbar), temperature (40–120°C) and applied voltage (400–600 V). The adjustable conditions of temperature, pressure and voltage determine the collision energy of the proton transfer reaction that usually operates at E/N = 130 Td (E being the electric field strength, and N being the number density of molecules present in the reaction chamber; 1 Td = 10^−17^ V cm^2^). All ions are extracted from the drift tube through a specially designed transfer lens system from where they are pulsed every 30 µs to the orthogonal time-of-flight region. All the pulsed ions are detected by a multiple-channel-plate (MCP, Burle Industries Inc., Lancaster, PA, USA) and separated according to their mass to charge (m/z) ratio. The resulting highly resolved mass spectra ranging between *m/z* = 20 and *m/z* = 315 were recorded every second. The raw PTR-TOF data are acquired by the TofDaq software (Tofwerk AG, Switzerland) and post processed by routines functions programmed in Matlab R2009b, 7.5 (The MathWorks Inc., Natick, MA, USA) [56]. Compounds of exactly known *m/z* such as 1,4 dichlorobenzene (*m/z* = 146.976) and 1,2,3 trichlorobenzene (*m/z* = 180.937) were continuously added to the sample inlet system through a diffusive cell and together with other known low mass ions were used for a precise conversion of “time-of-flight” into “mass-to-charge” ratio (*m/z)* in order to assign the exact mass scale and the sum formula of all ions during BVOC analysis.

All PTR-TOF signals were simultaneously detected with 1 s integration time. Background measurements were run before every set of experiments by sampling the empty cuvette and were always subtracted before BVOCs emission rates calculation. BVOC emission rates were normalized to either the extent of cut produced after leaf wounding or the leaf area exposed to light to dark transitions. The carbon emitted as BVOCs was calculated by multiplying the flux of BVOCs by the number of carbon atoms incorporated into the respective volatile molecule and then multiplied for the ratio between the carbon weight over the total weight of the molecule.

### Statistics

Measurements were carried out on at least four replicates (different leaves from different plants). The means were statistically separated using Tukey's post-hoc test. Different letters indicate statistically different means between groups (*P*<0.05). All statistical analyses were conducted using SigmaPlot 11.0 (SPSS; http://www.spss.com/).
